# circLMTK2 acts as a sponge of miR-150-5p and promotes proliferation and metastasis in gastric cancer

**DOI:** 10.1186/s12943-019-1081-4

**Published:** 2019-11-14

**Authors:** Sen Wang, Dong Tang, Wei Wang, Yining Yang, Xiaoqing Wu, Liuhua Wang, Daorong Wang

**Affiliations:** 1 Department of General Surgery, Northern Jiangsu People’s Hospital, Yangzhou University, Yangzhou, 225001 Jiangsu China; 2 Department of General Surgery, The First Affiliated Hospital of Nanjing Medical University, Nanjing, 210009 Jiangsu China; 3 Institute of General Surgery, Yangzhou University, Yangzhou, 225001 Jiangsu China; 4 GloriousMed Technology Co., Ltd., Shanghai, 200120 China; 5 Yangzhou Clinical Medical College of Nanjing Medical University, Yangzhou, 225001 Jiangsu China

**Keywords:** circLMTK2, Gastric cancer, miR-150-5p, C-Myc

## Abstract

**Background:**

As a novel class of non-coding RNAs, circular RNAs (circRNAs) are key regulators of the development and progression of different cancers. However, little is known about the function and biological mechanism of circLMTK2, also named hsa_circ_0001725, in gastric cancer (GC) tumourigenesis.

**Methods:**

circLMTK2 was identified in ten paired cancer specimens and adjacent normal tissues by RNA sequencing and genome-wide bioinformatic analysis and verified by quantitative real-time PCR (qRT-PCR). Knockdown or exogenous expression of circLMTK2 combined with in vitro and in vivo assays were performed to prove the functional significance of circLMTK2. The molecular mechanism of circLMTK2 was demonstrated by searching the CircNet database and confirmed by RNA in vivo precipitation assays, western blotting, luciferase assays and rescue experiments.

**Results:**

circLMTK2 was frequently upregulated in GC tissues, and high circLMTK2 expression was associated with poor prognosis, lymph node metastasis and poor TNM stage in GC patients. Functionally, circLMTK2 overexpression promoted GC cell proliferation and tumourigenicity in vitro and in vivo. Furthermore, ectopic circLMTK2 expression enhanced GC cell migration and invasion in vitro and tumour metastasis in vivo. In addition, we demonstrated that circLMTK2 could sponge miR-150-5p, thus indirectly regulating the c-Myc expression and contributing to GC tumourigenesis.

**Conclusion:**

Our findings demonstrate that circLMTK2 functions as a tumour promoter in GC through the miR-150-5p/c-Myc axis and could thus be a prognostic predictor and therapeutic target for GC.

## Introduction

Gastric cancer (GC) remains one of the most common and lethal malignancies worldwide, with a particularly high disease incidence rate in East Asia [1]. Although the incidences and mortality trends for GC have declined in recent years [2], the outcomes of this disease are still among the poorest of all solid-organ tumours, predominantly due to the frequent presence of advanced stage disease with lymphatic or distant metastasis [3]. Because there are limited therapeutic approaches for treating advanced GC, it is urgent that we search for novel biomarkers and prognostic indicators to reflect the disease status and develop more therapeutic targets for this lethal disease.

A poorly characterized component of the GC transcriptome is circular transcripts (circRNAs), which have been implicated in other diseases [4–6]. circRNAs are a type of RNA formed by back-splicing [7], and compared to their linear counterparts, they are highly stable due to their covalently closed loop [8]. Advances in high-throughput sequencing technology and novel bioinformatics algorithms have facilitated the systematic detection of circRNAs [9, 10]. circRNAs have been posited to function as sponges of microRNAs (miRNAs) and decoys of RNA-binding proteins (RBPs), which affect biological processes such as proliferation, metastasis, and apoptosis in tumour cells [11]. Therefore, circRNAs may be potential biomarkers or therapeutic targets [12].

In this study, we generated de-ribosome RNA sequencing data from GC tissues from ten patients, and identified approximately 35,350 circRNA candidates (at least two unique back-splicing reads). We characterized one circRNA derived from the LMTK2 gene locus, termed circLMTK2 (hsa_circ_0001725), that was upregulated in GC patients. In vitro and in vivo experiments showed that circLMTK2 can promote cell growth and metastasis. Moreover, a clinical analysis showed a significant negative correlation between circLMTK2 and the patient’s prognosis. Our study provides a new insight into the pathogenesis of GC.

## Materials and methods

### Human samples

We retrospectively collected ten paired cancer specimens and adjacent normal tissues from patients with gastric cancer who had surgically proven primary GC and received a D2 radical gastrectomy (R0 resection) at the Department of General Surgery of Northern Jiangsu People’s Hospital between November 2008 and December 2011. None of these patients received preoperative chemotherapy or radiotherapy. Clinicopathological features, which included age, sex, tumour site, tumour size, differentiation grade, TNM stage (American Joint Committee on Cancer classification, AJCC), lymphatic invasion and neural invasion, are shown in Table 1. The median follow-up time was 25.0 months (range: 1–85 months). The follow-up interval began on the date of surgery and ended on the date of disease progression, death or the last clinical investigation. This study was approved by the Medical Ethics Committee of Northern Jiangsu People’s Hospital. Written informed consent was obtained from all participants.

**Table 1:** Relationship between circLMTK2 expression and clinicopathologic factors of patients with gastric cancer

Parameter	No. of patients	circLMTK2 (high)	circLMTK2 (low)	*P* -value
Sex				0.871
male	67	35	32	
female	53	26	27	
Age (year)				0.965
< 60	46	24	22	
≥ 60	74	37	37	
Tumor site				0.681
Upper	26	12	14	
Middle	35	17	18	
Lower	47	24	23	
Diffuse	12	8	4	
Tumor size (cm)				0.279
< 5	54	24	30	
≥ 5	66	37	29	
Differentiation grade				0.734
Well-moderate	50	24	26	
Poor-undifferentiation	70	37	33	
Lauren classification				0.812
Intestinal	58	29	29	
Diffuse	37	20	17	
Mixed	20	9	11	
Uncertain	5	3	2	
T stage				0.017*
T1-T3	65	26	39	
T4	55	35	20	
Lymph node status				0.029*
Negative	56	22	34	
Positive	64	39	25	
Distant metastasis				0.227
M0	109	53	56	
M1	11	8	3	
TNM stage				0.004*
I-II	56	20	36	
III-IV	64	41	23	
Lymphatic invasion				0.446
Negative	51	29	22	
Positive	69	32	37	
Nervous invasion				0.694
Negative	52	28	24	
Positive	68	33	35	

The TNM Staging System is based on the tumor (T), the extent of spread to the lymph nodes (N), and the presence of metastasis (M)

* *P* < 0.05

### RNA-seq analysis

Total RNA was isolated using TRIzol reagent (Life Technologies, Carlsbad, CA, USA). Approximately 3 μg of total RNA from each sample was subjected to the RiboMinus Eukaryote Kit (Qiagen, Valencia, CA) to remove ribosomal RNA prior to RNA-seq library construction. Strand-specific RNA-seq libraries were prepared using a NEBNext Ultra Directional RNA Library Prep Kit for Illumina (NEB, Beverly, MA, USA). Briefly, approximately 50 ng of ribosome-depleted RNA samples was fragmented and then used for first- and second-strand cDNA synthesis with random hexamer primers. A dUTP mix was used for second-strand cDNA synthesis. An End-It DNA End Repair Kit was used to repair the ends of the double-stranded cDNA fragments, which were then modified by the Klenow fragment so that an A was added to the 3′ end of the DNA fragments and were finally ligated to adapters. The ligated products were purified and treated with uracil DNA glycosylase (UDG) to remove the second-strand cDNA. Purified first-strand cDNA was subjected to 13–15 cycles of PCR amplification, followed by library analysis with a Bioanalyzer 2100 (Agilent, Santa Clara, CA, USA); the cDNA was then sequenced using a HiSeq 2000 system (Illumina, San Diego, CA, USA) and a 100-bp paired-end run.

### Identification and quantification of circRNAs

The RNA-seq fastq reads were first mapped to the human reference genome (GRCh38/hg38) obtained from the UCSC genome database (http://genome.ucsc.edu/) using TopHat2. The unmapped reads were then used to identify circRNAs as previously described [5]. Briefly, the unmapped reads were processed to 20-nucleotide anchors from both ends of the sequencing read. Anchors that aligned in the reverse orientation (head-to-tail) represent a back-spliced junction. Anchor alignment was extended such that the complete read alignment and the breakpoint were flanked by a GT/AG splice site. The total number of reads that spanned back-spliced junctions was used as an absolute measure of circRNA abundance. The genomic regions that mapped to inferred circRNAs were annotated according to RefSeq. Gene coordinates were downloaded from the RefGene tables in the UCSC Genome Browser. The host genes of circRNAs were determined using a custom script. For each circRNA, we searched for the longest transcript fragment whose boundaries (5′ end or 3′ end) exactly matched both ends of the circRNA in the same strand and then defined the corresponding gene of the transcript fragment as the host gene of the circRNA.

### Cell culture and treatments

BGC-823 and AGS cells were cultured in RPMI 1640 supplemented with 10% foetal bovine serum (FBS) and 1% penicillin-streptomycin at 37 °C and 5% CO_2_. HEK 293 T and MGC-803 cells were cultured in Dulbecco’s modified Eagle’s medium (DMEM) supplemented with 10% FBS and antibiotics. Transcription was blocked by the addition of 2 μg/ml actinomycin D or DMSO (Sigma-Aldrich, St. Louis, MO, USA), which served as a control for the cell culture medium.

### RNA fluorescence in situ hybridization (FISH)

In situ hybridization was performed using specific probes to the circLMTK2 sequence. PCR fragments with the T7 promoter were amplified with specific primers for the back-splice region of circLMTK2. Primers are listed in Additional file 1: Table S3. Digoxin or biotin-labelled RNA probes were transcribed from PCR fragments using the DIG or biotin RNA labelling mix and T7 RNA polymerase (Roche) according to the manufacturers’ instructions. AGS cells were grown to the exponential phase and were 80–95% confluent at the time of fixation. After pre-hybridization (1 × PBS/0.5% Triton X-100), the cells were hybridized in hybridization buffer (40% formamide, 10% dextran sulfate, 1 × Denhardt’s solution, 4× SSC, 10 mM DDT, 1 mg ml^− 1^ yeast transfer RNA, 1 mg ml^−1^sheared salmon sperm DNA) with DIG-labelled probes specific to circLMTK2 at 60 °C overnight. Signals were detected using a tyramide-conjugated Alexa 488 fluorochrome TSA kit (Life Technologies). Nuclei were counterstained with 4,6-diamidino-2-phenylindole. Images were acquired on a Leica SP5 confocal microscope (Leica Micosystems, Mannheim, Germany).

### circRNA in vivo precipitation (circRIP)

A biotin-labelled circLMTK2 probe (5′-CTACCTGTTTGACCAGGGTCTCTGGGTGT-3′-biotin) was designed and synthesized by RiboBio (Guangzhou, China), and a circRIP assay was performed as described. circLMTK2-overexpressing BGC-823 cells were seeded in a 10-cm dish. After reaching sufficient confluency, the cells were transfected with the specific biotin-tagged probe or control probe at a final concentration of 200 nmol/L. Then, the cells were fixed with 1% formaldehyde for 10 min, lysed and sonicated. After centrifugation, 50 μl of the supernatant was retained as input, and the remaining cell lysis solution was incubated with a circLMTK2-specific probe-streptavidin-dynabead (M-280, Invitrogen) mixture overnight at 30 °C. The next day, the M-280 dynabead-probe-circRNA mixture was washed and incubated with 200 μl lysis buffer and proteinase K to reverse the formaldehyde crosslinking. Finally, total RNA was extracted from the mixture using an miRNeasy Mini Kit according to the manufacturer’s instructions (Qiagen).

### Nucleic acid preparation and quantitative real-time polymerase chain reaction (qRT-PCR)

Genomic DNA was isolated with a QIAamp DNA Mini Kit (Qiagen, Valencia, CA, USA), and total RNA was isolated using TRIzol reagent (Life Technologies, Carlsbad, CA, USA).The nuclear and cytoplasmic fractions were isolated using NE-PER Nuclear and Cytoplasmic Extraction Reagents (Thermo Scientific). Total RNA from the nuclear and cytoplasmic fractions was isolated with TRIzol. Complementary DNA was synthesized using a PrimeScript RT reagent kit (Takara Bio Inc., Dalian, China), and RT-PCR was performed using SYBR Premix Ex Taq (Takara Bio Inc.). For miRNA measurements, mature miR-150-5p was reverse-transcribed and quantified with TaqMan® RT primers and probes, and the data were normalized to U6 small nuclear RNA expression using predesigned TaqMan assays (Applied Biosystems, Foster City, USA). The primers are listed in Additional file 1: Table S3.

### Vector construction

The circLMTK2 genomic region and its wild-type flanking introns were amplified from the cDNA using PrimerSTAR Max DNA Polymerase Mix (Takara) and were subcloned into a pcDNA3.0 vector. In the luciferase reporter assay, circLMTK2 was amplified from the cDNA and was inserted into the region directly downstream of a cytomegalovirus (CMV) promoter-driven firefly luciferase cassette in the pCDNA3.0 vector. The 3′-UTR sequence of c-Myc was amplified and inserted into the psiCHECK-2 vector (Promega, Madison, USA). Mutations in the miRNA binding sites in the c-Myc 3′-UTR sequence were generated using a Mut Express II Fast Mutagenesis Kit (Vazyme, NanJing, China). The constructs were verified by sequencing. The primers are listed in Additional file 1: Table S3.

### Oligonucleotide transfection

SiRNA and miRNA mimics and inhibitors were synthesized by RiboBio (Guangzhou, China). The sequences that were used are shown in Additional file 1: Table S3. The cells were transfected using Lipofectamine RNAiMax (Life Technologies).

### Luciferase reporter assay

HEK 293 T cells (5 × 10^3^) were seeded into 96-well plates and were cotransfected with a mixture of 50 ng of psiCHECK-2 vector, and miRNA mimics. After 48 h of incubation, the firefly and Renilla luciferase activities were quantified with a dual-luciferase reporter assay (Promega, Madison, WI, USA).

### Transwell assay

A Matrigel Cell Migration Assay and Invasion System was used to measure cell migration and invasion in vitro as described previously. For cell migration assays, AGS, MGC-803 and BGC-823 cells (5 × 10^4^ cells/well) were suspended in 50 μL serum-free RPMI 1640 and DMEM and placed in the upper collagen-coated chambers (8-mm pore size; Millipore, Temecula, CA, USA) of each transwell insert (Matrigel: serum-free medium 1:5) respectively. Next, 800 μL of media with 10% FBS was placed in the lower chamber and incubated for 24 and 48 h. After incubation, cells adhering to the upper surface of the membrane were removed with a cotton swab. The migrated cells that adhered to the lower surface were fixed with 4% formaldehyde in PBS, followed by staining with a 1% crystal violet solution. All samples were then examined and photographed under light microscopy at × 200. The total cells were counted, and the total percentage of inhibition based on the cells in each image was measured as described previously. For the invasion assays, which were performed almost the same as the cell migration assays, Matrigel was used instead of collagen on the filter membrane as described previously.

### 5-Ethynyl-2′-deoxyuridine (EdU) incorporation assay

EdU assays were performed with a Cell-Light EdU DNA Cell Proliferation Kit (RiboBio, Shanghai, China). Cells (1 × 10^4^) were seeded in each well of 96-well plates. After incubation with 50 μM EdU for 2 h, the cells were fixed in 4% paraformaldehyde and stained with Apollo Dye Solution. Hoechst 33342 was used to stain the nucleic acids within the cells. Images were obtained with an Olympus FSX100 microscope (Olympus, Tokyo, Japan), and the number of EdU-positive cells was counted.

### CCK8 assay

Cell proliferation was assessed by Cell Counting Kit-8 assays (Dojindo Laboratories, Kumamoto, Japan). Cells (1 × 10^3^) were seeded into 96-well plates. Then, 10 μl of CCK-8 solution was added to each well on days 1, 2, 3, 4 and 5. After 2 h of incubation at 37 °C, the absorbance at 450 nM was measured using an automatic microplate reader (Synergy4; BioTek, Winooski, VT, USA). The experiment was repeated three times.

### Colony formation assay

For the colony formation assays, cells were trypsinized, and 1 × 10^3^ cells were plated in 6-well plates and incubated at 37 °C for 14 days. Colonies were dyed with a 0.1% crystal violet and 20% methanol solution. Cell colonies were then counted and analysed.

### Animal experiments

BGC-823 cells that stably expressed circLMTK2 and control cells were harvested and suspended in DMEM without FBS. Sixteen mice (male BALB/c-nu/nu, 6 weeks old) were divided randomly into two groups, and each mouse was injected subcutaneously in the lower back with 2 × 10^6^ cells in 200 μl of DMEM without FBS. The mice were monitored weekly for tumour weight and tumour volume (volume = length ×width^2^/2). At approximately 4 weeks after injection, the mice were sacrificed, and the tumours were dissected and weighed. For the in vivo tumour metastasis studies, eighteen mice (male BALB/c-nu/nu, 6 weeks old) were divided randomly into two groups. BGC-823 cells stably transfected with circLMTK2 lentiviruses or control vector were injected into the lateral tail veins of the nude mice (2 × 10^6^ cells per mouse). Fifty days later, the mice were sacrificed and examined for the numbers of lung metastatic colonies. Paraffin sections were imaged with a Leica Microsystems Microscope (Leica Biosystems, Wetzlar, Germany). The mouse experiments were conducted according to the Guide for the Care and Use of Laboratory Animals of Yangzhou University. The protocol was approved by the Committee on the Ethics and Welfare of Laboratory Animal Science of Yangzhou University.

### Statistical analysis

Statistical analyses were performed using SPSS 20.0 (IBM, SPSS, Chicago, IL, USA) and GraphPad Prism for Windows, version 6.00 (GraphPad Software, La Jolla, USA). Unless otherwise stated, Student’s t-test and one-way ANOVA were used to determine the statistical significance for comparisons of 2 or more groups. The Pearson correlation coefficient was used to analyse the correlations. Overall survival (OS) was measured from the date of surgery. For OS, patients known to be alive at the time of the last follow-up were censored on the last date of contact. OS curves were calculated with the Kaplan-Meier method and were analysed with the log-rank test. Univariate analysis and multivariate models were constructed using a Cox proportional hazards regression model. *P* values < 0.05 were considered statistically significant.

## Results

### Identification of circular RNAs by RNA-seq in GC

**Fig. 1:**
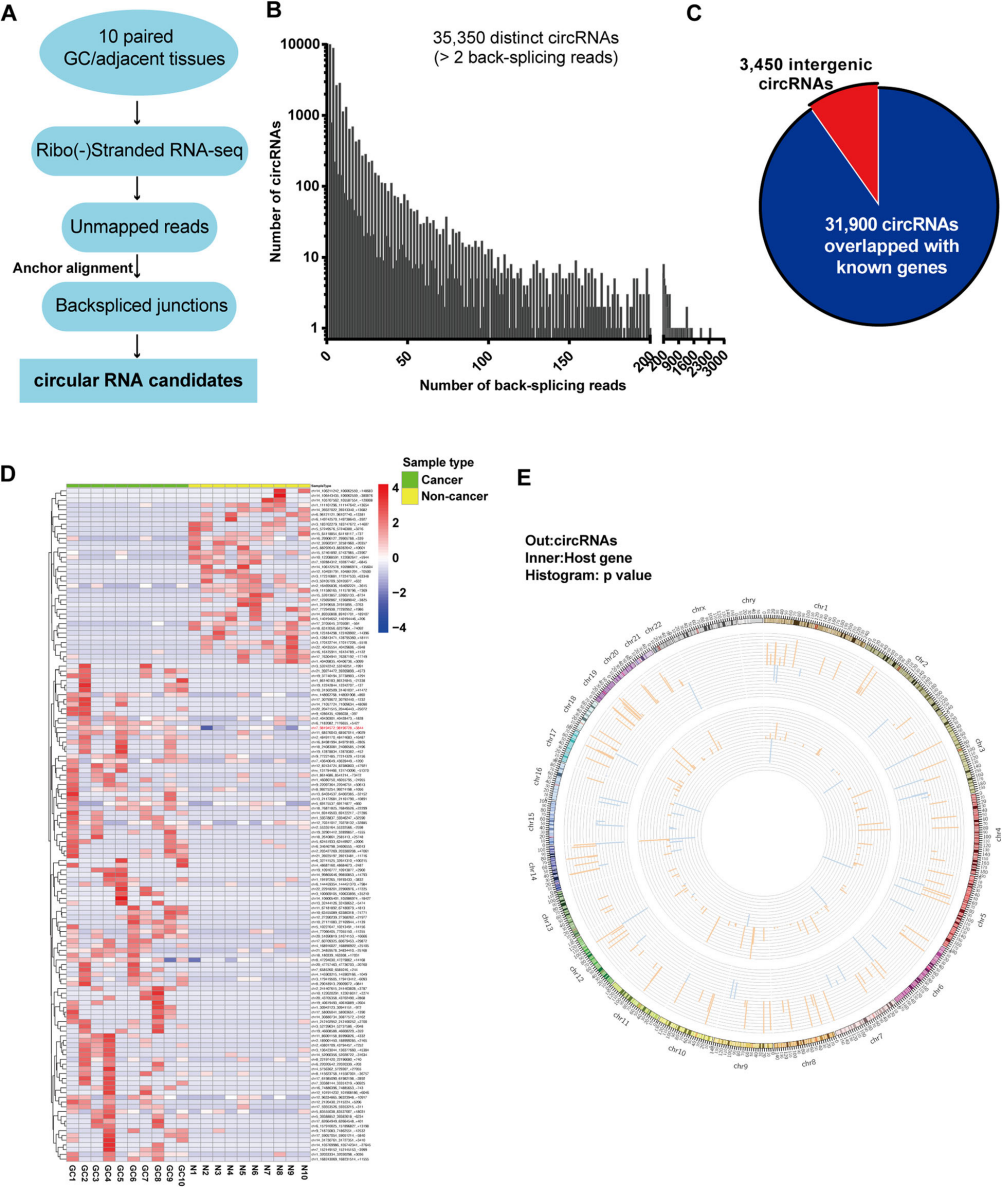
Identification of circular RNAs by RNA-seq analyses in GC. (**a**) RNA-seq analysis of circular RNAs in ten paired human GC tissues and matched normal tissues. (**b**) Total number of circRNAs and back-spliced reads that were identified in ten paired GC tissues and matched normal tissues. (**c**) Origin of circRNAs in the genome. (**d**) Clustered heatmap of the differentially expressed circRNAs in ten paired human GC tissues and matched normal tissues. Rows represent circRNAs, while columns represent tissues. The circRNAs were classified according to the Pearson correlation. (**e**) Circo plots showing differentially expressed circRNAs and their host genes in GC tissues. Outer: differentially expressed circRNAs; Inner: host genes. The values represent the log (fold change) of cancer vs. normal, red: upregulated, blue: downregulated

We first characterized circular RNA transcripts using an RNA-seq analysis of de-ribosome RNA-seq from ten paired GC/adjacent tissues (Fig. 1a). Each sample was sequenced on an Illumina HiSeq and yielded ~ 60 million reads, which were mapped to the human reference genome (GRCh38/hg38) by TopHat2 [13]. A computational pipeline based on the anchor alignment of unmapped reads was used to identify circRNAs without reliance on gene annotations [5]. Collectively, 35,350 distinct circRNA candidates, which contained at least two unique back-splicing reads, were found in these tissues (Fig. 1b, Additional file 2: Table S1). Among these circRNAs, there are 3450 intergenic circRNAs, and 31,900 overlapped with known genes (Fig. 1c). The expression analysis of these circRNA transcripts revealed that a series of circRNAs was differentially expressed in cancerous tissues compared with those in matched normal tissues. Among the 142 differentially expressed circRNAs (filtered by |FC(fold change)| ≥ 2 and *P*<0.05), 105 were upregulated, and 37 were downregulated in GC compared with those in normal tissues (Fig. 1d, Additional file 3: Table S2). These circRNAs and their host genes were located in diverse genomic regions (Fig. 1e). Then, we further confirmed the RNA-seq results of four circRNAs in 25 paired normal and cancerous gastric tissues by qRT-PCR analysis (Additional file 1: Figure S1).

### Characterization of circLMTK2 in GC

**Fig. 2:**
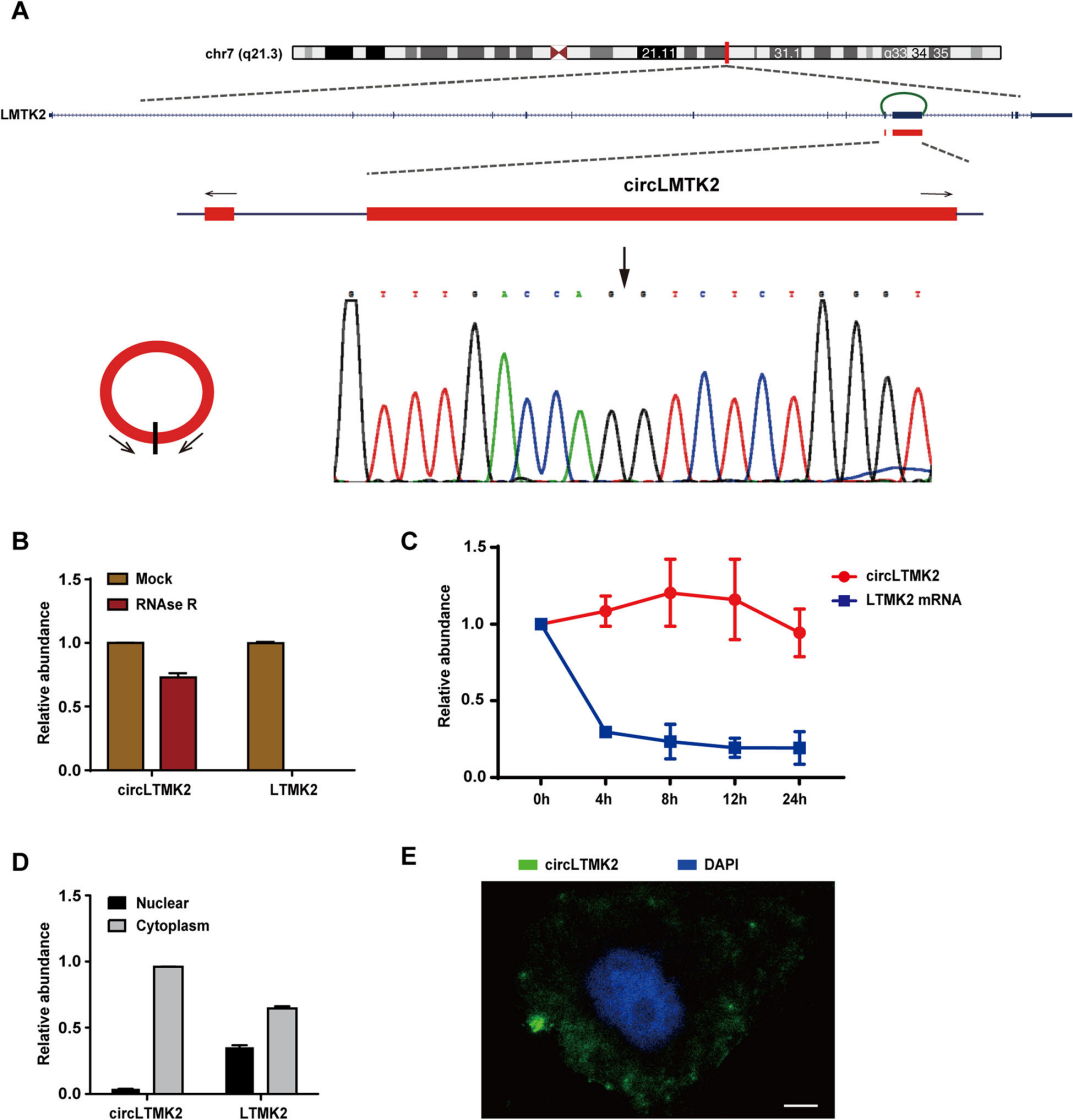
Characteristics of the circLMTK2 in GC cells. (**a**) The genomic loci of the LMTK2 gene and circLMTK2. The expression of circLMTK2 was detected by qRT-PCR and was validated by Sanger sequencing. The arrows represent divergent primers that bind to the genomic region of circLMTK2. (**b**) qRT-PCR analysis of circLMTK2 and LMTK2 mRNA after treatment with RNase R. (**c**) qRT-PCR analysis of circLMTK2 and LMTK2 mRNA after treatment with actinomycin D at the indicated time points. (**d**) qRT-PCR analysis of circLMTK2 and LMTK2 mRNA in either the cytoplasm or the nucleus. (**e**) RNA fluorescence in situ hybridization (FISH) for circLMTK2. The nuclei were stained with 4,6-diamidino-2-phenylindole (DAPI). Scale bar, 5 μm

We noted that one of the most differentially expressed circRNAs (chr7:98190727–98,194,572, GRCh38/hg38, Fig. 2a) was derived from a protein-coding gene locus, LMTK2, located on chromosome 7q21.3. Thus, we termed this circRNA as “circLMTK2” (*hsa_circ_0001725*). The genomic structure shows that circLMTK2 is looped by the tenth and eleventh exons of the LMTK2 gene (*NM_014916*). We designed outward-facing primers (Additional file 1: Table S3) and confirmed the junction site of circLMTK2 by Sanger sequencing (Fig. 2a). Resistance to digestion by RNase R exonuclease further confirmed that this RNA species exists in a circular form (Fig. 2b). Total RNA was harvested at the indicated time points after treatment with actinomycin D, a transcription inhibitor. Analysis of circLMTK2 and LMTK2 mRNA revealed that the circRNA isoform circLMTK2 is highly stable, as its transcript half-life exceeded 24 h, whereas the linear transcript of LMTK2 mRNA exhibited a half-life of < 4 h in AGS cells (Fig. 2c). Next, qRT-PCR analysis of nuclear and cytoplasmic RNA and FISH against circLMTK2 demonstrated that the circular form of LMTK2 preferentially localized within the cytoplasm in AGS cells (Fig. 2d and e). These results suggest that circLMTK2 is a stable and cytoplasmic circRNA derived from the LMTK2 mRNA.

### circLMTK2 promotes GC cell proliferation and tumourigenicity in vitro and in vivo

**Fig. 3:**
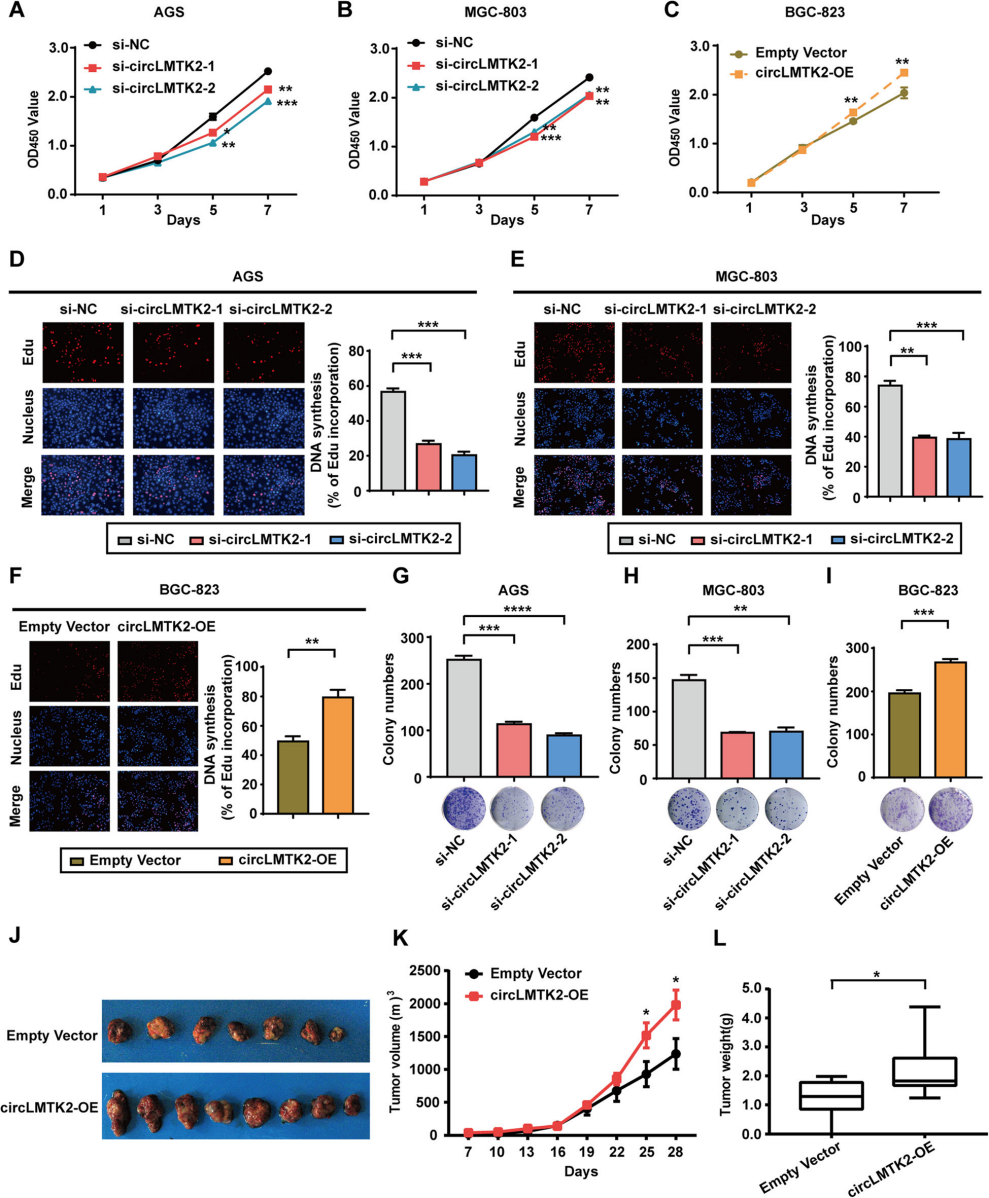
circLMTK2 promotes GC cell proliferation and tumourigenicity in vitro and in vivo*.* (**a** and **b**) Assessment of the proliferation of AGS and MGC-803 cells transfected with control or circLMTK2 siRNAs by CCK-8 assay. (**c**) Stable circLMTK2 overexpression promoted BGC-823 cell proliferation. OD, optical density. (**d** and **e**) Assessment of DNA synthesis using an EdU assay in MGC-803 and AGS cells transfected with control or circLMTK2 siRNAs. (**f**) Stable circLMTK2 overexpression promoted DNA synthesis in BGC-823 cells. Micrographs represent at least three experiments. Scale bar = 200 μm. (**g** and **h**) Colony formation assay using AGS and MGC-803 cells transfected with control or circLMTK2 siRNAs. (**i**) Stable circLMTK2 overexpression promoted BGC-823 cell colony formation. (**j**) Xenograft assay with BGC-823 stable cell lines. (**k**) circLMTK2 over-expression increased the volume of the xenograft tumours. (**l**) circLMTK2 over-expression increased the weight of the xenograft tumours. (**a**-**l**) Results are shown as the mean ± standard error of the mean (SEM) of three experiments. *P < 0.05; **P < 0.01; ***P < 0.001 (Student’s t-test)

To investigate the potential role of circLMTK2 in GC cells, we initially explored the effect of blocking circLMTK2 on cell growth. We designed two siRNAs to target the back-splice sequence. As expected, siRNA directed against the back-splice sequence inhibited only the circular transcript of circLMTK2 and did not affect the expression of the LMTK2 linear species (Additional file 1: Figure S3C). Silencing circLMTK2 expression significantly suppressed cell proliferation rates (Fig. 3a and b) and colony formation abilities in both GC cell lines (Fig. 3g and h) and impaired nucleotide synthesis (Fig. 3d and e). In contrast, the ectopic expression of circLMTK2 in BGC-823 cells (designated circLMTK2-OE), which was induced by lentiviruses with circular frames and circLMTK2 sequences (Additional file 1: Figure S3A and B), dramatically promoted cell growth (Fig. 3c and i) and increased the EdU incorporation rate (Fig. 3f). Furthermore, circLMTK2-OE and empty vector cells were inoculated subcutaneously into the flanks of nude mice, and these mice were monitored closely for tumour growth for 4 weeks. Our results illustrated that tumours derived from circLMTK2-OE cells were significantly larger than those derived from empty vector cells, both in terms of tumour volumes and weights (Fig. 3j–l). These results suggest that circLMTK2 significantly promotes GC cell proliferation and tumourigenicity in vitro and in vivo.

### circLMTK2 enhances GC cell migration and invasion in vitro and tumour metastasis in vivo

**Fig. 4:**
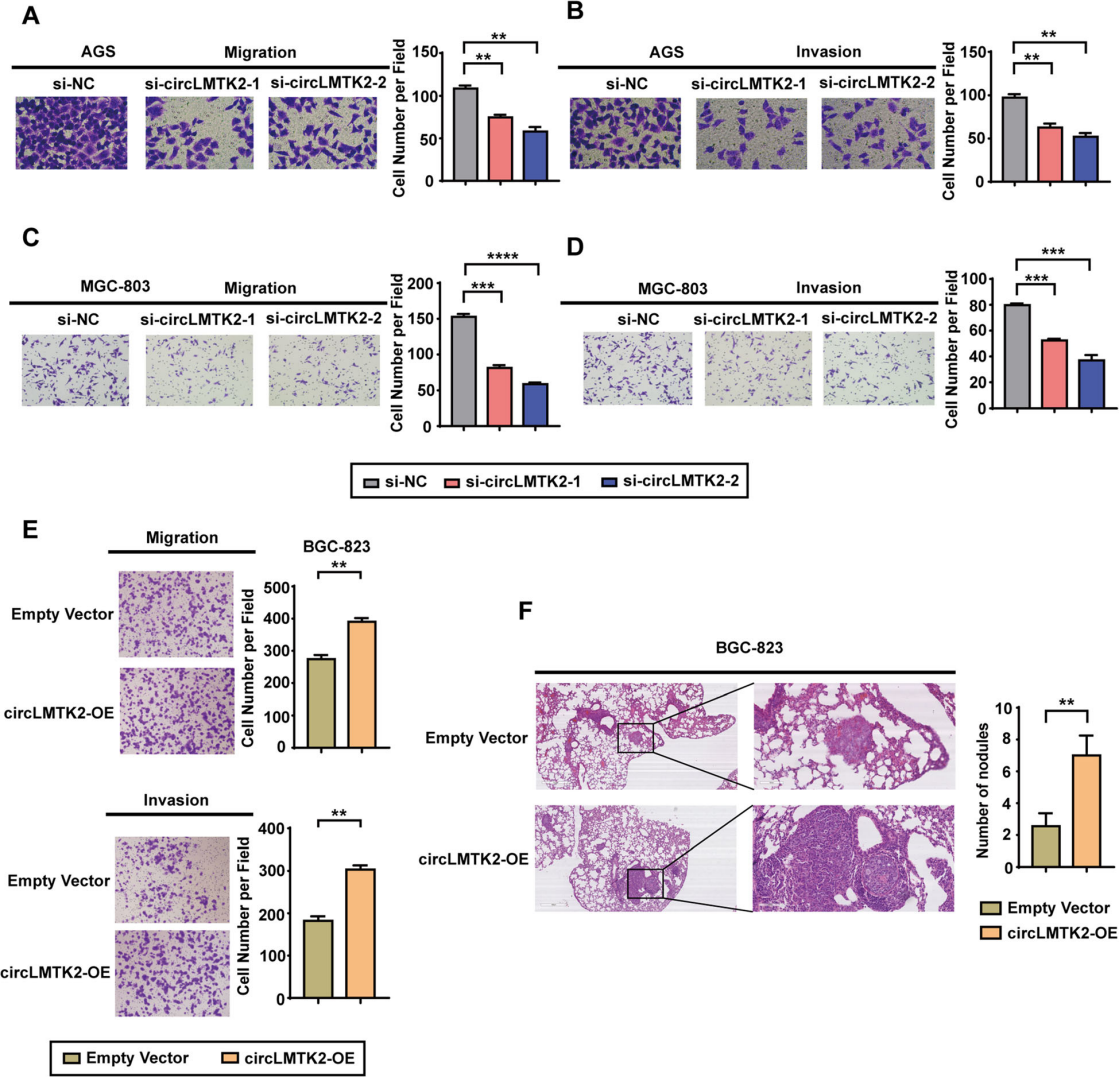
circLMTK2 enhances GC cell migration and invasion in vitro and tumour metastasis in vivo. (**a** and **b**) Silencing circLMTK2 inhibits the migration and invasion of AGS cells transfected with control or circLMTK2 siRNAs. (**c** and **d**) Silencing circLMTK2 inhibits the migration and invasion of MGC-803 cells transfected with control or circLMTK2 siRNAs. (**e**) Stable circLMTK2 overexpression promoted BGC-823 cell migration and invasion in vitro. Representative images are shown on the left. The values shown on the right represent the mean ± SEM. (**f**) BGC-823 cells stably transfected with circLMTK2 or empty vector control were injected into the tail vein of BALB/c nude mice (2 × 10^6^ cells per mouse, *n* = 9 for each group). The nude mice treated with circLMTK2-transfected cells demonstrated significantly more lung metastatic colonies. The data are presented as the mean ± SEM. Scale bars, 100 and 400 μm. The paraffin sections were imaged with a Leica Microsystems Microscope (Leica biosystems, Wetzlar, Germany). (A-F) The data are the means ± SEM of three experiments. *P < 0.05; **P < 0.01; ***P < 0.001 (Student’s t-test)

Next, we studied the effects of circLMTK2 expression on GC cell migration and invasion. Our migration and invasion assay results showed that circLMTK2 knockdown significantly eliminated the migratory and invasive capacities of AGS and MGC-803 cells when compared with the indicated controls conditions (Fig. 4a-d). In contrast, the migration and invasion were greater in BGC-823 cells overexpressing circLMTK2 than in empty vector cells (Fig. 4e). These results indicate that circLMTK2 increases cell invasion and metastasis, which we further validated in vivo. The in vivo metastasis assay was performed by injecting circLMTK2-OE and empty vector cells into nude mice through the lateral tail vein to examine the cells’ lung metastasis ability. Metastatic nodules in the lungs were confirmed histologically. The number of metastatic nodules was significantly higher in mice injected with circLMTK2-OE BGC-823 cells than in mice injected with empty vector BGC-823 cells (Fig. 4f). Consistently, these data demonstrate that circLMTK2 significantly promotes GC cell migration and invasion in vitro and tumour metastasis in vivo.

### circLMTK2 abundantly sponges miR-150-5p in GC cells

**Fig. 5:**
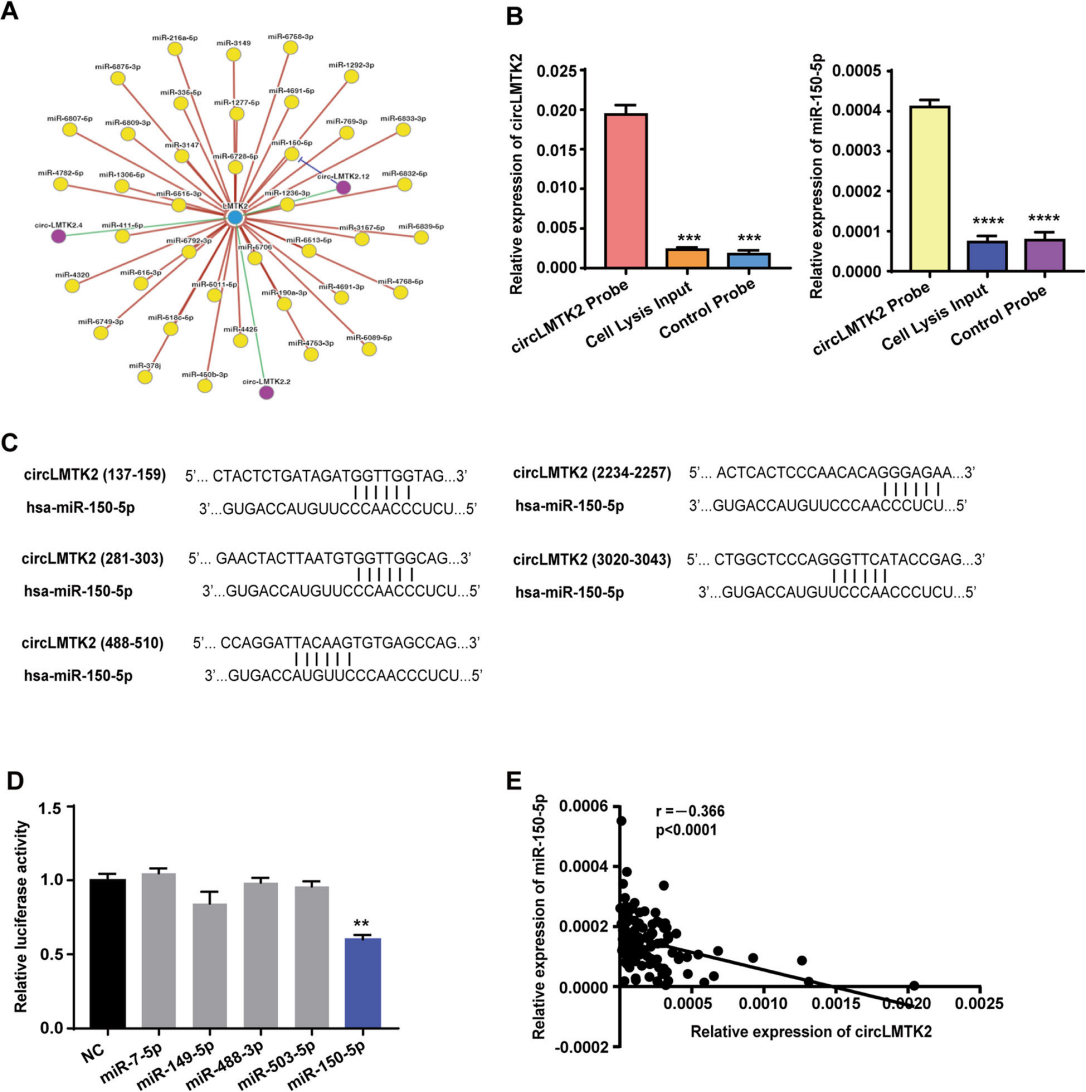
circLMTK2 serves as a miRNA sponge for the miR-150-5p. (**a**) circRNA-miRNA-gene regulatory network from the CircNet database. (**b**) Left: circLMTK2 in GC cell lysate was pulled down and enriched with a circLMTK2- specific probe and then detected by qRT-PCR. Right: miR-150-5p was pulled down and enriched with a circLMTK2-specific probe and then detected by qRT-PCR. (**c**) A schematic drawing shows the putative binding sites of miR-150-5p with respect to circLMTK2. (**d**) A luciferase reporter assay was used to detect the luciferase activity of LUC-circRNA in HEK 293 T cells cotransfected with miRNA mimics. The data are the means ± SEM of three experiments. (**e**) The correlation between circLMTK2 and miR-150-5p levels in 120 GC patients. Pearson’s correlation coefficient values (r) and *P* values are as indicated

Given that circRNA has been shown to act as a miRNA sponge and that circLMTK2 is abundant and stable in the cytoplasm, we next investigated the ability of circLMTK2 to bind to miRNAs. CircNet (http://syslab5.nchu.edu.tw/CircNet/) and TargetScan (http://www.targetscan.org) were used to predict the potential target miRNAs that could bind with the circLMTK2 sequence (Fig. 5c), and miR-150-5p was selected as the best potential target of circLMTK2 (Fig. 5a). Next, we designed a specific biotin-labelled circLMTK2 probe to perform RNA in vivo precipitation (RIP) to confirm whether miR-150-5p can interact with circLMTK2 in GC cells, which has been reported in several studies [14–16]. We purified circLMTK2-associated RNA and performed qPCR to measure circLMTK2 and miR-150-5p expression. The results showed a significant enrichment of circLMTK2 and miR-150-5p compared to the controls (Fig. 5b), indicating that miR-150-5p was sponged by circLMTK2 in GC cells. We further constructed a circLMTK2 fragment and inserted it immediately downstream of the luciferase reporter gene (LUC+ circLMTK2). Then, miR-150-5p or some potential miRNAs mimics were transfected with reporter gene into HEK 293 T cells. A significant reduction in luciferase reporter activity was observed relative to co-transfection with control mimic or other miRNAs (Fig. 5d). In addition, there was a significant inverse correlation between miR-150-5p and circLMTK2 expression levels in 120 GC samples (Fig. 5e). Together, these results suggest that circLMTK2 may serve as a binding platform for miR-150-5p.

### circLMTK2 promotes GC cell growth and metastasis by sponging miR-150-5p and downregulating c-myc

**Fig. 6:**
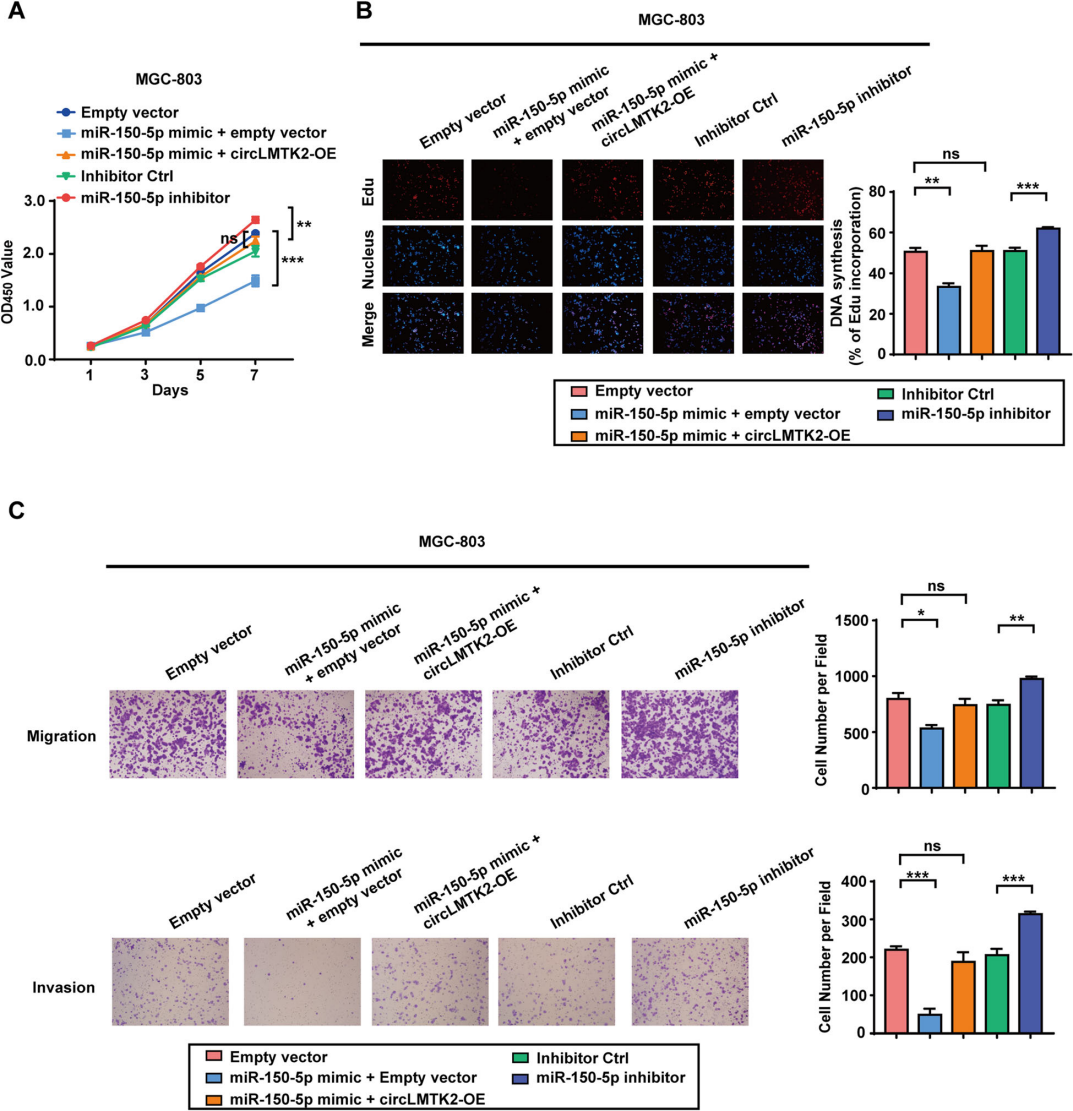
circLMTK2 promotes GC cell growth and metastasis by sponging miR-150-5p (**a**) The re-introduction of circLMTK2 reversed the inhibitory effect of miR-150-5p on MGC-803 cell proliferation. MiR-150-5p inhibitors promoted MGC-803 cell proliferation. (**b**) The re-introduction of circLMTK2 reversed the inhibitory effect of miR-150-5p on DNA synthesis. MiR-150-5p inhibitors promoted GC cell DNA synthesis. The micrographs represent at least three experiments. Scale bar = 200 μm. (**c**) The suppressed migration of MGC-803 cells induced by miR-150-5p was restored via circLMTK2 re-introduction. MiR-150-5p inhibitors promoted GC cell migration and invasion in vitro. (**a**-**c**) The data are the means ± SEM of three experiments. *P < 0.05; **P < 0.01; ***P < 0.001; ****P < 0.0001 (Student’s t-test)

Previous studies have described the tumour suppressor role of miR-150-5p in various cancers [17–19]; however, the potential mechanisms of miR-150-5p regulation of GC progression remain unclear. We detected that miR-150-5p overexpression in MGC-803 cells blocked the proliferation rate (Fig. 6a), DNA synthesis (Fig. 6b), and migration and invasion abilities (Fig. 6c), while miR-150-5p blockade had the opposite effects on GC cells. To further address whether circLMTK2 executes its function by interacting with miR-150-5p, we cotransfected miR-150-5p mimics and circLMTK2 expression constructs into GC cells. The effects on GC cell growth and motility suppression induced by miR-150-5p were reversed when the cells overexpressed circLMTK2 (Fig. 6a-c and Additional file 1: Figure S5A-D). These data reveal that circLMTK2 promotes GC cell growth and metastasis by sponging miR-150-5p.

**Fig. 7:**
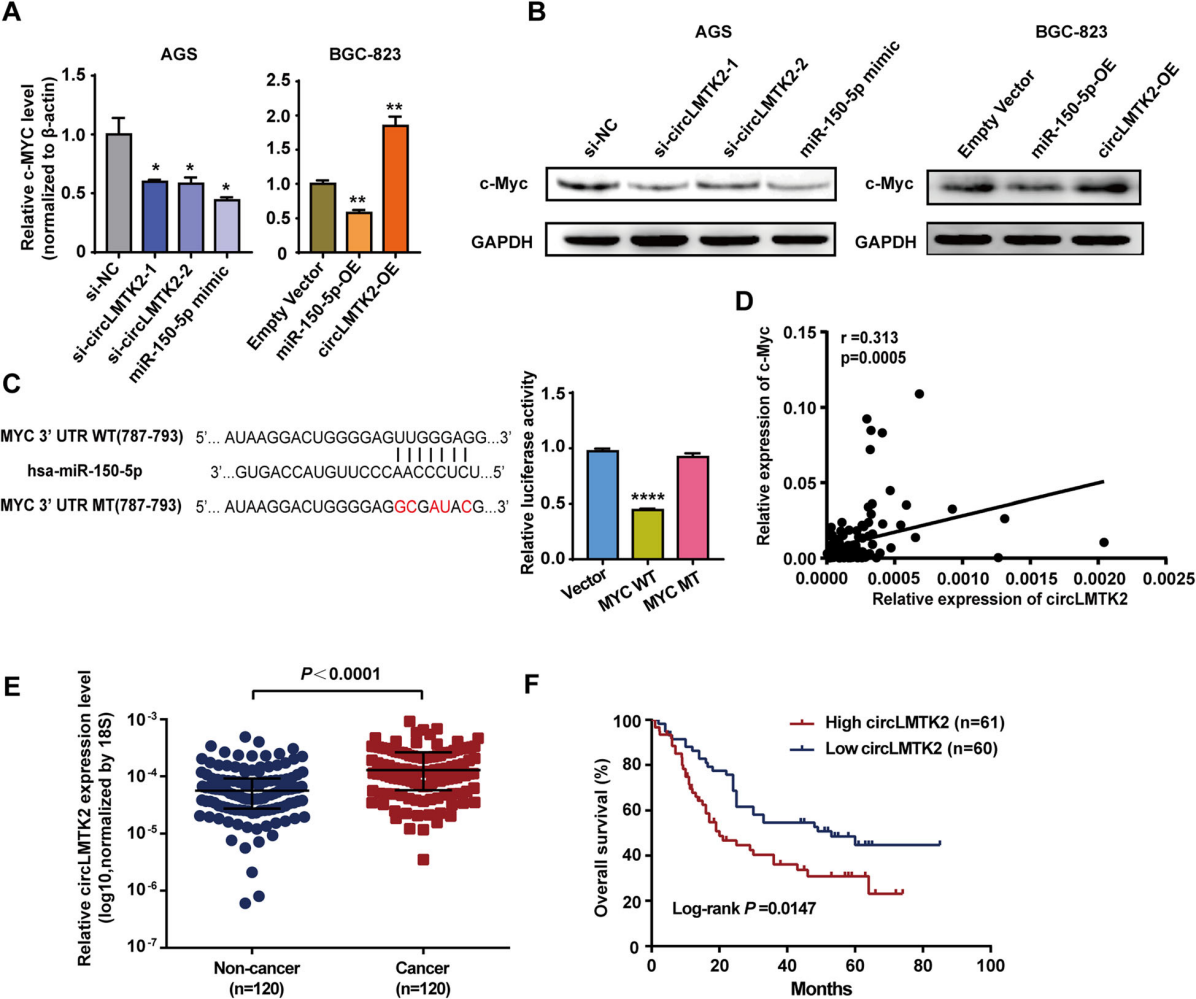
circLMTK2 expression is upregulated in GC. (**a**) c-Myc mRNA levels were repressed by silencing circLMTK2 or miR-150-5p overexpression in GC cells. In contrast, c-Myc mRNA levels were enhanced by circLMTK2 overexpression in GC cells. The data are the means ± SEM of three experiments. *P < 0.05; **P < 0.01 (Student’s t-test) (**b**) c-Myc protein levels were repressed by silencing circLMTK2 or miR-150-5p overexpression in GC cells. In contrast, c-Myc protein levels were enhanced by circLMTK2 overexpression in GC cells. (**c**) Left: Diagram of putative miR-150-5p binding sites in the 3′-UTR of c-Myc. The mutant sequences c-Myc 3′-UTR sequences used in the luciferase reporter constructs are indicated in red. Right: Relative activities of luciferase reporters containing c-Myc 3′-UTR variants cotransfected with miR-150-5p or negative control mimics in HEK 293 T cells. ****P < 0.0001 (Student’s t-test). (**d**) Correlation between circLMTK2 and c-Myc levels in GC tissues. Pearson’s correlation coefficient values (r) and P values are as indicated. (**e**) Relative circLMTK2 mRNA levels in 120 matched human GC/normal tissues. The values are expressed as medians with interquartile ranges. (**f**) Kaplan-Meier analysis of the correlation between circLMTK2 expression and overall survival (OS) in 120 patients with GC. Log-rank tests were used to determine statistical significance. The patients were divided into two groups according to the median value of circLMTK2 expression

We previously studied that c-Myc-associated circular RNAs, and we noted that circLMTK2 overexpression could increase c-Myc expression at both the mRNA and protein levels, while circLMTK2 knockdown and miR-150-5p overexpression exerted the opposite effects on GC cells (Fig. 7a-b). As expected, we validated that c-Myc could be a potential target gene of miR-150-5p using the TargetScan database. 3′-UTR luciferase reporter assays further confirmed that miR-150-5p could bind directly to a site in the 3′-UTR of c-Myc (Fig. 7c). Although a correlation analysis showed a weak positive correlation between circLMTK2 expression and c-Myc mRNA levels in cancerous tissues (Fig. 7d), it also indicated that circLMTK2 could indirectly regulate c-Myc expression to some extent. These results suggested that c-Myc might be a putative target of miR-150-5p.

### circLMTK2 expression is upregulated in GC

To assess the clinical impact of the circLMTK2 expression on GC, we collected clinical data from the aforementioned patients. As presented in Fig. 7e, circLMTK2 levels were significantly higher in tumour tissues than in non-cancerous tissues. More importantly, increased circLMTK2 expression in GC tissues was significantly correlated with poor prognosis in GC patients, as shown by the Kaplan-Meier survival curve using the median value of circLMTK2 expression as the cut-off value (median survival of 16 months vs 45 months, *P* = 0.0114, log-rank test; Fig. 7f). Significantly higher numbers of late T stage (T4) or poor TNM stage (III-IV) tumours and cases of positive lymph node status were found in the high-circLMTK2 group (*P* < 0.05, Table 1). No significant differences were observed in the other clinical and pathological characteristics between the high and low circLMTK2 groups. We also performed univariate and multivariate Cox proportional hazards analyses, which included several known prognostic markers (sex, age, tumour size, differentiation grade, TNM stage, Lauren classification, lymphatic invasion and nerve invasion). The results showed that circLMTK2 level and TNM stage were independent prognostic factors for OS in patients with GC (Table 2).

**Table 2:** Univariate analysis identifies factors influencing the overall survival rate of gastric cancer patients

Factors	Univariate analysis		Multivariate analysis	
	HR (95% CI)	*P* value	HR (95% CI)	*P* value
Sex	1.454 (0.812–1.784)	0.188		
Age	1.243 (0.864–1.790)	0.241		
Tumor size	1.332 (0.995–2.034)	0.108		
Differentiation grade	0.823 (0.691–1.032)	0.156		
Lauren classification	1.096 (0.563–1.872)	0.038*		0.148
TNM stage	1.586 (1.012–3.986)	0.001*	2.043 (1.640–4.232)	0.002*
Lymphatic invasion	1.825 (1.256–2.771)	0.024*		0.204
Nervous invasion	1.643 (1.157–2.635)	0.013*		0.192
circLMTK2 expression	0.736 (0.033–1.326)	0.001*	0.832 (0.354–1.012)	0.001*

*HR* Hazard ratio, *CI* confidence interval

* *P* < 0.05

## Discussion

Recently, with the advent of next-generation sequencing, numerous circular RNAs have been identified from various animal genomes. Many of these highly stable circRNAs are abundantly expressed and play a role in many diseases, especially in tumours, via acting as miRNA sponges, decoying proteins, and affecting translation [20]. RNA-seq results provide useful information for revealing general circRNA expression trends and helping select candidate circRNAs for further research. In this study, we successfully identified thousands of circRNAs in human GC tissues and normal gastric tissues, and hundreds of them were differentially expressed. Nevertheless, the expression of each candidate circRNA still needs to be verified in a cohort of clinical samples and cell lines because most of the circRNAs have low abundance (Additional file 1: Figure S2).

circRNAs have been characterized as vital factors in cancer biology [21, 22]. It was previously reported that circRNAs could act as a miRNA “sponge” to decrease the abundance of miRNAs [23]. Shen et al. conducted a study showing that circRNA_001569 could significantly increase cell viability and inhibit cell apoptosis in GC via the miR-145/NR4A2 axis [24]. Another research group found that the novel circRNA-100,269 could target miR-630, leading to the inhibition of tumour cell growth [25]. circRNA_0023642 was shown to induce apoptosis and suppress cell proliferation, migration and invasion in GC via the EMT signalling pathway [26]. Additionally, circLARP4 was found to sponge miR-424, thus inhibiting the biological behaviours of GC [27]. Several studies revealed that circRNAs might also be valuable factors for the diagnosis of GC. Li et al. have assessed the plasma circRNA_002059 levels and determined a qRT-PCR method for detecting circRNA levels in GC patients [28]. Lower levels of circRNA_0000190 were also found in 104 gastric patient plasma samples and served as a novel non-invasive diagnostic biomarker for GC [29]. One recent study showed that GC cell-derived exosomes could promote the transformation of preadipocytes into brown-like cells by delivering ciRS-133 to suppress miR-133 and activate PRDM16 [30]. These studies show that the primary function of circRNAs is to deregulate miRNAs in pathogenic processes, which is important for demonstrating their potential role in cancer [31].

Here, we demonstrated that circLMTK2, which is an exonic circRNA originating from exons 10 and 11 of LMTK2 mRNA, was upregulated in GC tissues and predominantly localized in the cytoplasm. circLMTK2 is derived from the human lemur tyrosine kinase 2 gene, which is known as LMTK2. Previous studies have focused mainly on the biological function of LMTK2 in prostate cancer. The rs6465657 variant of LMTK2 was evidently related to the development of prostate cancer [32]. Additionally, LMTK2 has been identified as a driver mutation in lung adenocarcinoma by a large-scale RNA-seq analysis [33].. Recently, He et al. reported that circLMTK2 could act as a novel tumour suppressor in GC; however, they did not explain the source of circLMTK2 or submit any RNA-seq data in their study [34]. In addition, we validated the expression of circLMTK2 in our 25-patient cohort by qRT-PCR using He’s primer (Additional file 1: Table S3). We further confirmed that circLMTK2 was indeed upregulated in tumours compared with that in non-cancerous tissues (Additional file 1: Figure S4). Further analyses were conducted to determine the biological function and molecular mechanism of circLMTK2 in GC cells. We found that circLMTK2 promoted GC cell proliferation and tumourigenicity in vitro and in vivo. Furthermore, ectopic circLMTK2 expression effectively enhanced GC cell migration and invasion in vitro and tumour metastasis in vivo.

circRNAs have been suggested to function as miRNA sponges. We also demonstrated that circLMTK2 could bind to miR-150-5p and that five miR-150-5p-binding elements in circLMTK2 are essential for their interaction. MiR-150 has been implicated as either an oncogene or a tumour suppressor in various types of solid tumours [35–38]. MiR-150-5p was first considered as the main miRNA in immune and haematopoietic cells [39]. It has recently been shown that Myc upregulates LIN28 expression in myeloid cells, which inhibits miR-150 maturation from its precursor pri-miR-150 [40]. However, the function of miR-150-5p in the pathogenesis of GC is unknown. We also demonstrated that miR-150-5p inhibited GC cell proliferation and motility in vitro. More importantly, we identified that circLMTK2 exerts oncogenic functions by sponging miR-150-5p.

Intriguingly, we found that c-Myc is a potential functional target of miR-150-5p. c-Myc is a key basic helix-loop-helix leucine zipper transcription factor that is frequently upregulated in various human cancers, including GC [41]. Its overexpression contributes to malignant transformation by regulating the expression of a number of genes participating in multiple aspects of tumourigenesis, such as cell cycle progression, cell invasion, migration, metastasis, and angiogenesis [42–46]. c-Myc has been implicated in controlling miRNA expression, and c-Myc-regulated miRNAs affect virtually all aspects of the hallmarks of Myc-driven diseases [47, 48]. Increasing evidence has indicated that there is significant crosstalk between c-Myc and miRNAs. In fact, c-Myc regulates the expression of a number of miRNAs, resulting in widespread miRNA repression, and c-Myc is regulated by miRNAs, leading to sustained Myc activity [49]. Our results showed that circLMTK2 could regulate c-Myc expression by sponging miR-150-5p, indicating that c-Myc may act as an essential component of the regulatory circuit and providing further mechanistic evidence to support the notion that c-Myc is a promising therapeutic target in the treatment of GC.

In conclusion, our study reveals that circLMTK2 is upregulated in GC tissues and that high circLMTK2 expression is associated with poor prognosis, lymph node metastasis and poor TNM stage in GC patients. Moreover, enhanced circLMTK2 expression promoted GC cell growth and motility in vitro and in vivo through sponging miR-150-5p to upregulate c-Myc. Consequently, circLMTK2 may have considerable potential as a prognostic predictor and therapeutic target for GC.

## Supplementary information

**Additional file 1: Table S3.** Primers and RNA sequences used in this study. **Figure S1.** Validation of 4 differentially expressed circRNA candidates in GC tissues using qRT-PCR in 25 paired GC tissues and matched normal gastric tissues. **Figure S2.** Expression levels of circLMTK2 in 8 GC cell lines. **Figure S3.** Schematic illustration of the circLMTK2 overexpression vector. **Figure S4.** Detection of circLMTK2 in GC tissues by qRT-PCR using He’s primer in 25 paired GC tissues and matched normal gastric tissues. **Figure S5.** circLMTK2 promotes GC cell growth and metastasis by sponging miR-150-5p.

**Additional file 2: Table S1.** 35,350 distinct circRNA candidates.

**Additional file 3: Table S2.** 142 differentially expressed circRNAs in GC compared with those in normal tissues.

## Data Availability

All data generated or analysed during this study are included in this published article and its Additional files.

## Abbreviations

CCK-8: Cell Counting Kit-8
circRIP: CircRNA in vivo precipitation
circRNA: Circular RNA
c-Myc: MYC proto-oncogene, bHLH transcription factor
EdU: 5-Ethynyl-20-deoxyuridine
FISH: Fluorescence in situ hybridization
GC: Gastric cancer
LMTK2: Lemur tyrosine kinase 2
miRNA: MicroRNA
OE: Overexpression
OS: Overall survival
qRT-PCR: Quantitative real-time polymerase chain reaction
