# Supplementary material for: circLMTK2 acts as a sponge of miR-150-5p and promotes proliferation and metastasis in gastric cancer
Source: Mol Cancer. 2019 Nov 14;18:162. doi: 10.1186/s12943-019-1081-4 (PMC6854648; doi:10.1186/s12943-019-1081-4)
Supplement: Supplementary file 1 — Additional file 1: Table S3. Primers and RNA sequences used in this study. Figure S1. Validation of 4 differentially expressed circRNA candidates in GC tissues using qRT-PCR in 25 paired GC tissues and matched normal gastric tissues. Figure S2. Expression levels of circLMTK2 in 8 GC cell lines. Figure S3. Schematic illustration of the circLMTK2 overexpression vector. Figure S4. Detection of circLMTK2 in GC tissues by qRT-PCR using He’s primer in 25 paired GC tissues and matched normal gastric tissues. Figure S5. circLMTK2 promotes GC cell growth and metastasis by sponging miR-150-5p. [file 12943_2019_1081_MOESM1_ESM.doc]

**Supplementary Figures**


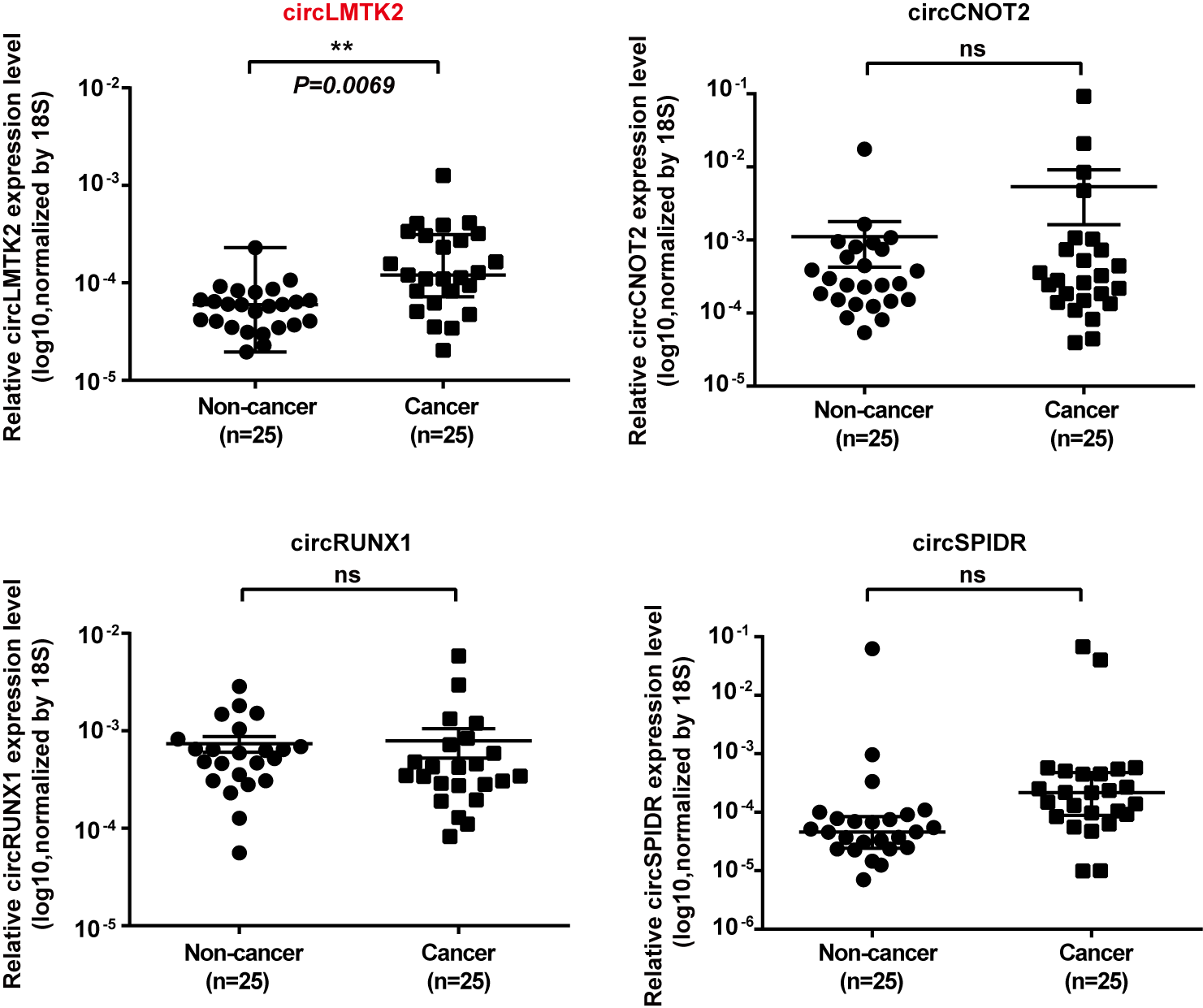


**Fig. S1 Validation of 4 differentially expressed circRNA candidates in GC tissues using qRT-PCR in 25 paired GC tissues and matched normal gastric tissues. The values are expressed as medians with interquartile ranges.* P < 0.05; ** P < 0.01. Ns, not significant**


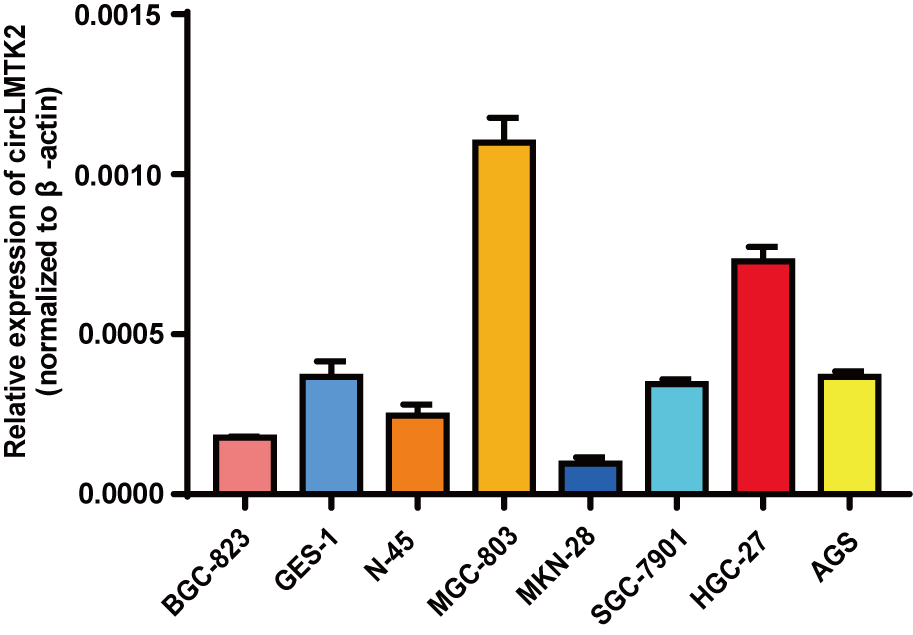


**Fig. S2 Expression levels of circLMTK2 in 8 GC cell lines.**


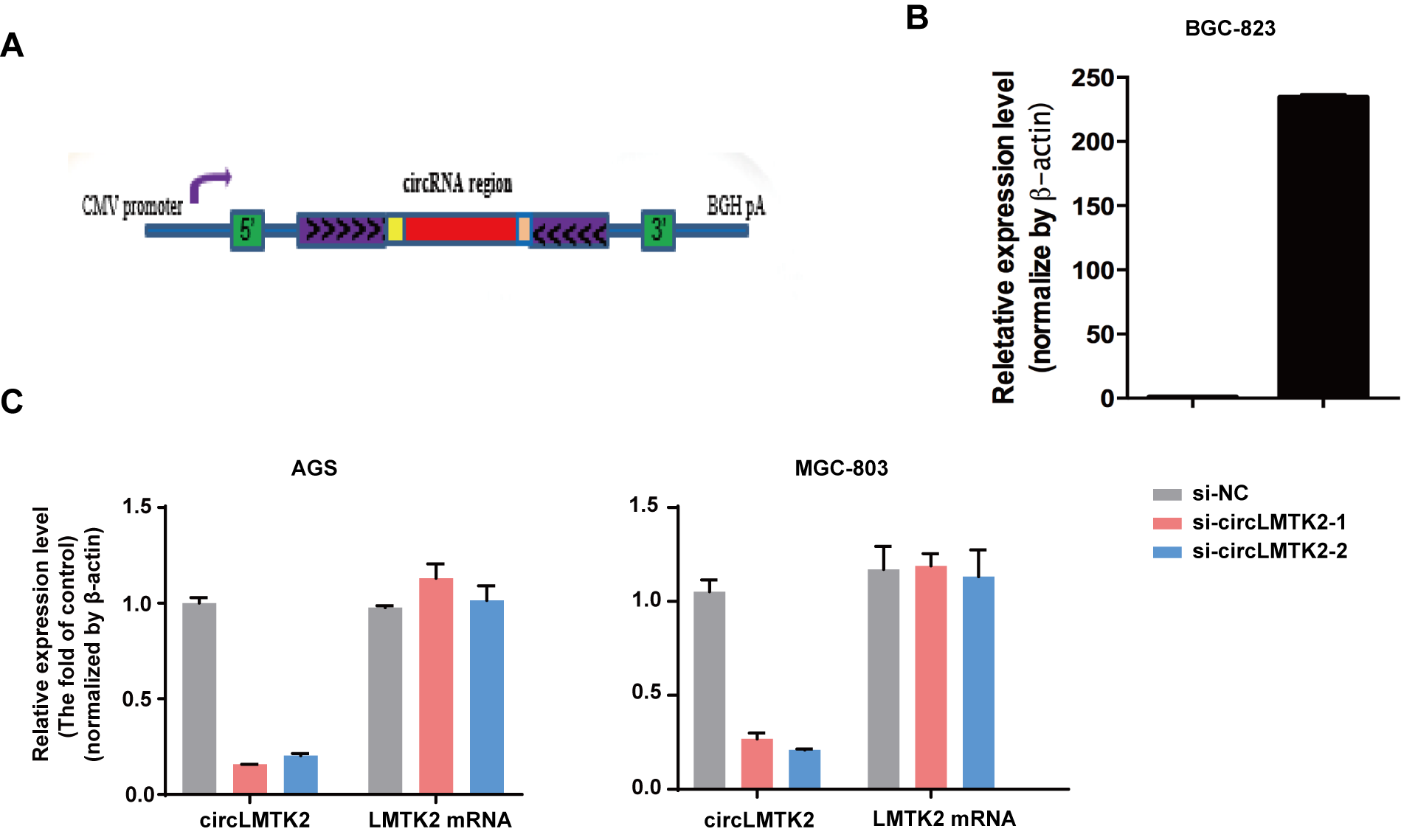


**Fig. S3 (A) Schematic illustration of the circLMTK2 overexpression vector. (B) Detection of circLMTK2 overexpression in the circLMTK2-OE stable BGC-823 cell line. (C) qRT-PCR analysis of circLMTK2 and LMTK2 mRNA expression after treatment with two siRNAs.**


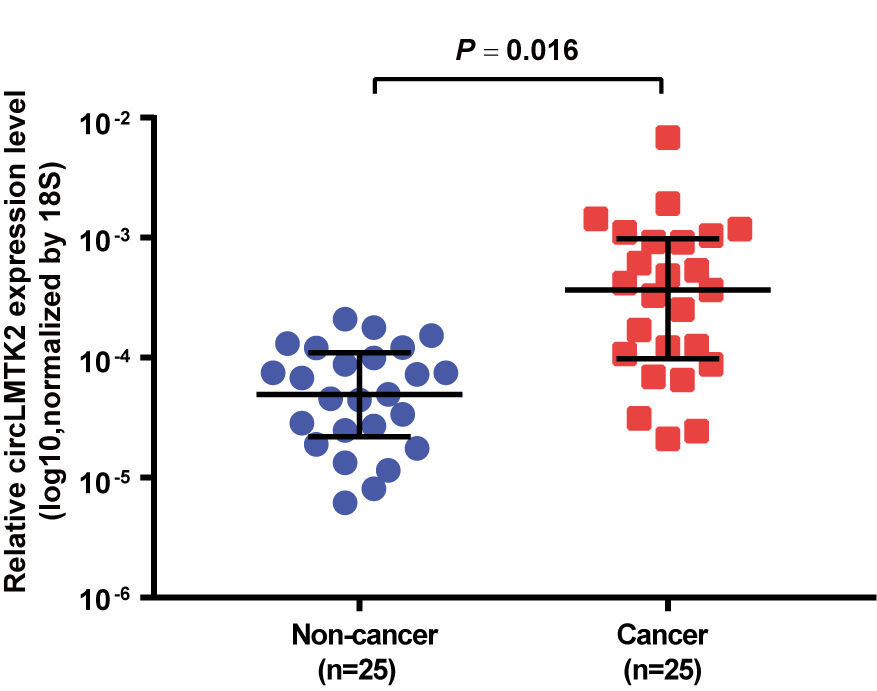


**Fig. S4 Detection of circLMTK2 in GC tissues by qRT-PCR using He’s primer in 25 paired GC tissues and matched normal gastric tissues. The values are expressed as medians with interquartile ranges.**


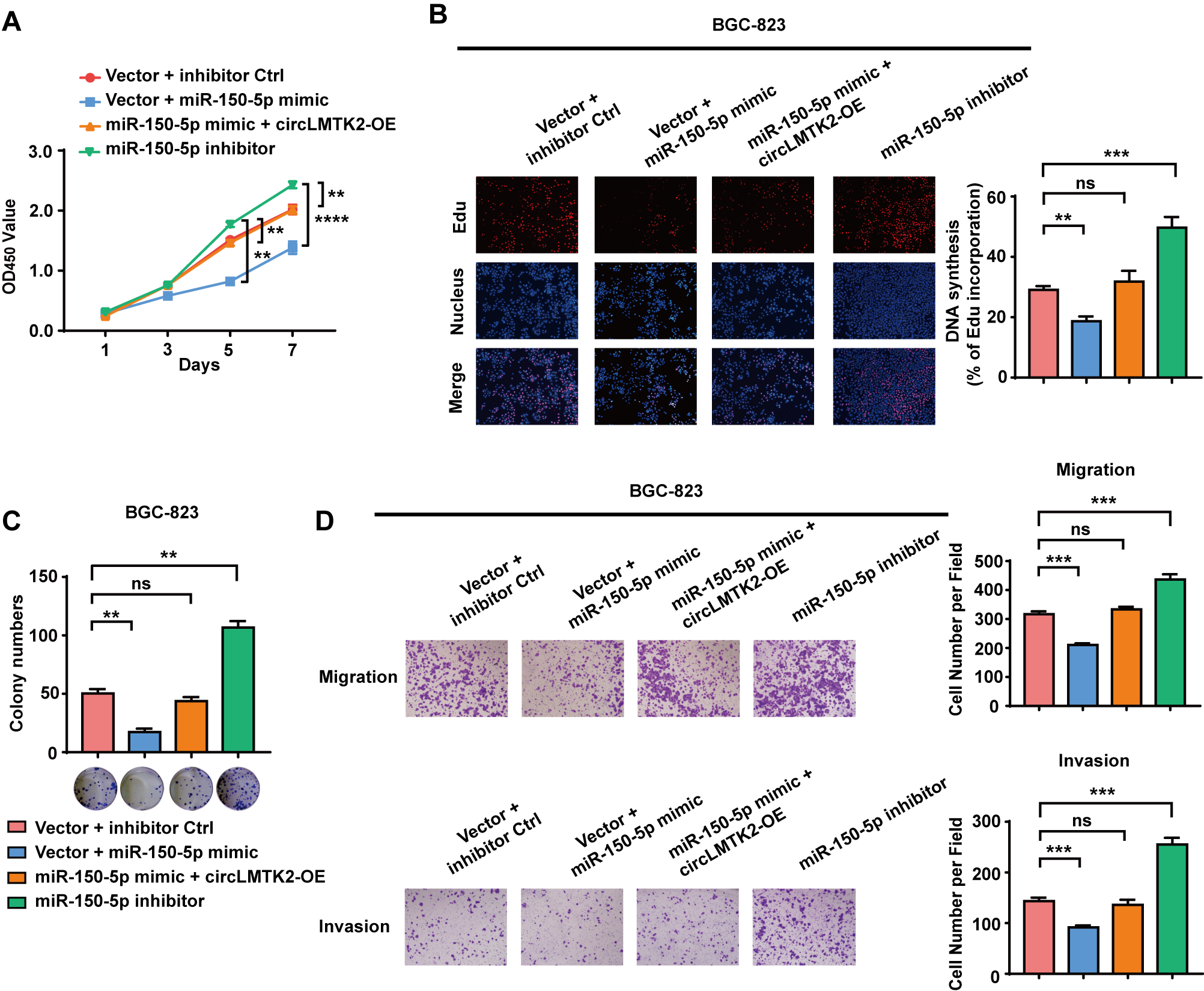


**Fig. S5 circLMTK2 promotes GC cell growth and metastasis by sponging miR-150-5p**

**(A) The re-introduction of circLMTK2 reversed the inhibitory effect of miR-150-5p on cell proliferation. MiR-150-5p inhibitors promoted GC cell proliferation. (B) The re-introduction of circLMTK2 reversed the inhibitory effect of miR-150-5p on DNA synthesis. MiR-150-5p inhibitors promoted GC cell DNA synthesis. The micrographs represent at least three experiments. Scale bar =200 μm. (C) The re-introduction of circLMTK2 reversed the inhibitory effect of miR-150-5p on colony formation. MiR-150-5p inhibitors promoted GC cell colony formation. Representative images are shown on the bottom. (D) The suppressed migration of GC cells induced by miR-150-5p was restored via circLMTK2 re-introduction. MiR-150-5p inhibitors promoted GC cell migration and invasion in vitro. (A-D) The data are the means ± SEM of three experiments. *P < 0.05; **P < 0.01; ***P < 0.001; ****P < 0.0001 (Student’s t-test).**

**Supplementary Table**

**Supplementary Table S3 Primers and RNA sequences used in this study**

| **List of oligonucleotide sequences** | **5'--> 3'** |
| --- | --- |
| **primers for Real-time PCR and RT-PCR** | |
| circLMTK2-qF | CGAGGACTGGAAGAAGGA |
| circLMTK2-qR | GGAAAGGTTTGAATACGG |
| LMTK2-qF | AACTGTGTATCCTGCTGTAAGG |
| LMTK2-qR | CTGCTGGTGGTGTGAAATCTA |
| Actin-qF | TTGTTACAGGAAGTCCCTTGCC |
| Actin-qR | ATGCTATCACCTCCCCTGTGTG |
| 18S-qF | TTAATTCCGATAACGAACGAGA |
| 18S-qR | CGCTGAGCCAGTCAGTGTAG |
| circSPIDR-F | GAAGATGACGAGGGTGCTGT |
| circSPIDR-R | CCTGCAGTGGTCTTTCTCCT |
| circCNOT2-F | TCCACATCGGTCAGAAAAAGAT |
| circCNOT2-R | GAGGCAATCAAATAGAACTGTCACT |
| circRUNX1-F | ATCCGTGGTTCCAAGTGTC |
| circRUNX1-R | GGTGTCCCAATAGGTCTGC |
| circLMTK2-He-qF | GGAAGAAGGAAAAGAAGGC |
| circLMTK2-He-qR | TGGAAAGGTTTGAATACGC |
|  | |
| **Oligos for plasmid construction** | |
| circLMTK2-exp-vector-F | gcagaattcGGTCTCTGGGTGTGACACTTTGG |
| circLMTK2-exp-vector-R | gcaggtaccCTGGTCAAACAGGTAGACTGTGA |
| LUC-cricLMTK2-F | CCTCGAGGTTTAAACTACGGGGTCTCTGGGTGTGACACTTTGG |
| LUC-cricLMTK2-R | ATCATATGACTAGTCCCGGGCTGGTCAAACAGGTAGACTGTGA |
|  | |
| **FISH probes** | |
| FISH-dig- F | TTTCGATGATGTCACAGTCT |
| FISH-dig-T7-R | TAATACGACTCACTATAGGGAGAGGAAAGGTTTGAATACGG |
|  | |
|  | |
| **primers for site-directed mutation** | |
| LUC-MYC-UTR-WT-F | CCTCGAGGTTTAAACTACGGGGAAAAGTAAGGAAAACGATTCC |
| LUC-MYC-UTR-WT-R | ATCATATGACTAGTCCCGGGTCAAAGAAAGTAATTATTTATTG |
|  |  |
| **siRNAs** | |
| si-circLMTK2-1 | CUGUUUGACCAGGUCUCUG |
| si-circLMTK2-2 | ACCUGUUUGACCAGGUCUC |
|  |  |
